# Management of Chronic Health Situations

**DOI:** 10.3390/healthcare14020176

**Published:** 2026-01-09

**Authors:** Luís Sousa, Christoph Käppler, Fabiana Faleiros, Geyslane Alburquerque, Helena José

**Affiliations:** 1 Nursing Department, Atlântica School of Health, Atlantic University, 2730-036 Barcarena, Portugal; hjose@uatlantica.pt; 2 RISE—Health Research Network, University of Porto, 4200-319 Porto, Portugal; 3 Comprehensive Health Research Centre, University of Évora, 7005-345 Évora, Portugal; 4 Faculty of Rehabilitation Sciences, University of Dortmund, 44227 Dortmund, Germany; christoph.kaeppler@tu-dortmund.de; 5 Ribeirão Preto College of Nursing, University of São Paulo, Ribeirao Preto 14040-902, Brazil; fabifaleiros@eerp.usp.br; 6 Department of Nursing, Federal University of Pernambuco, Recife 50670-901, Brazil; geyslane.melo@ufpe.br; 7 Health Sciences Research Unit, Nursing, Coimbra Nursing School, 3000-232 Coimbra, Portugal

Chronic diseases are one of the greatest current global health challenges, not only because of their direct clinical impacts, but above all because of the functional, emotional, and social repercussions that profoundly affect the lives of patients and their families [1,2]. The comorbidities associated with these conditions lead to significant limitations, requiring continuous symptom management and permanent adaptations in daily life, often provided by informal caregivers. These caregivers, an essential pillar of health systems, face substantial physical and emotional overload, as demonstrated across various cultural and socioeconomic contexts [1,2,3].

Therefore, understanding the transitions associated with chronic disease, as well as self-management and rehabilitation processes, is essential to ensure more equitable and sustainable health responses aligned with the Sustainable Development Goals, particularly SDG 3 [4].

Despite advances in chronic disease management, significant gaps remain in the integration of person-centered approaches, health literacy, safe transitions of care, and long-term rehabilitation into daily practice. Evidence remains fragmented on how digital skills, self-care support, and caregiver inclusion can be systematically operationalized in different care settings. In addition, inequalities in access to care, particularly among vulnerable populations and in low- and middle-income settings, continue to limit the effectiveness of current chronic care models [5,6,7,8,9].

In this Special Issue, we seek to highlight the importance of person-centered care, as recommended in the Registered Nurses’ Association of Ontario (RNAO) guide [10], and of safe transitions throughout the disease pathway [11].

The five articles [12,13,14,15,16] presented herein approach this topic by analyzing emerging phenomena and proposing interventions that strengthen autonomy, health literacy, and the well-being of people with chronic diseases.

To begin the collection, Lee and Shim’s [12] article explores the influence of diabetes knowledge and attitudes toward Internet health information on e-Health literacy in middle-aged adults with diabetes, highlighting the importance of digital literacy and a proactive attitude in the autonomous management of chronic diseases. This research demonstrates that higher levels of knowledge and positive attitudes are associated with greater e-health literacy, underscoring the need for nursing interventions that foster informational skills as an integral part of clinical self-care.

Previously published evidence indicates that higher levels of online health literacy are associated with greater diabetes-related knowledge and enhanced self-care behaviours, mediated by increased self-efficacy [17,18,19,20].

In the field of psychosocial determinants of chronicity, Lorber et al. [13] address the relationship between loneliness, frailty, and mental health in people with chronic diseases. The results show significant associations between frailty, feelings of social isolation, and deterioration in psychological well-being, pointing to the need for therapeutic and community strategies that address social and emotional dimensions in addition to traditional biomedical management.

Studies on loneliness underscore the importance of investing in self-management programs for chronic illness, as these interventions contribute to reductions in loneliness and mitigate its adverse effects on health and well-being [21,22,23].

Fleischhauer et al. [14] provide a methodologically distinct contribution, outlining the pilot implementation of a disease management concept in primary health care for people with venous leg ulcers. The study highlights the usefulness of case management approaches and education focused on both the individual and the care team, promoting their greater involvement and that of the family in the therapeutic process and generating positive perceptions about the use of recommended therapies.

These findings are consistent with previous studies showing that patients’ understanding of the etiology of venous leg ulcers and the mechanism of action of compression therapy is associated with greater adherence to treatment [24,25,26,27]. In the context of managing chronicity associated with neurological conditions. Ref. [15] analyzed urinary incontinence in people with spinal cord injury and neurogenic bladder, exploring its relationship with satisfaction and lifestyle. The results show that urinary incontinence remains a frequent complication, with a significant impact on quality of life, autonomy, and social participation. This study reinforces the importance of individualized approaches that integrate appropriate methods of bladder emptying, therapeutic education, and continuous monitoring, recognizing people with spinal cord injury as active participants in the management of their chronic condition.

Evidence in this area supports the effectiveness of clean intermittent catheterization and recommends that healthcare professionals assist in the selection of appropriate materials and patient education, in order to optimize technique, manage complications and prevent adverse outcomes, including sediment and lithiasis, urinary incontinence, urethral lesions and strictures, and recurrent urinary tract infections [28,29,30].

In the area of barriers to accessing specific treatments, the article by Venkatesh et al. [16] examines obstacles to accessing antiviral therapy for hepatitis C in low- and middle-income countries. By identifying factors such as underdiagnosis, insufficient infrastructure, and stigmatization, the article reinforces the role of robust public health policies and educational initiatives in ensuring that effective interventions reach the most vulnerable populations—an imperative for health equity.

The main barriers to the treatment of viral hepatitis in low- and middle-income countries are consistent to those reported in previous studies, with stigma identified as a central determinant [31,32].

This Special Issue brings together contributions that illustrate the multiplicity of factors—informational, psychosocial, organizational, and structural—that shape the experience of chronicity and the responses of health systems. The published articles emphasize the centrality of an integrated, person-centered approach that goes beyond isolated biomedical management to include health literacy, psychosocial support, coordination between services, and overcoming barriers to care access. This collection reinforces the importance of sustainable care models that respond to the complex needs and responses of people with chronic diseases, promoting not only better clinical outcomes but also a higher quality of life and active participation in their health care. This Issue thus constitutes a contribution to the body of knowledge that drives evidence-informed practices, models of care integration, and health policies that respond to the imperatives of the 2030 Agenda for sustainable and inclusive care [33,34,35]. We urge readers to explore the various perspectives presented here, recognizing that the management of chronic health conditions is a dynamic, multifaceted field that is essential to the future of health care globally.

Future research should prioritize longitudinal studies focused on the implementation of person-centered approaches that examine the sustainability of these interventions, the role of digital health in supporting self-management, and structured support models for informal caregivers [36,37,38,39]. Greater emphasis should also be placed on co-creation approaches involving patients and family members, as well as policy-oriented research that links clinical evidence to health system transformation. By addressing these priorities, health research in general, and nursing research in particular, can contribute even more to equitable, safe, and sustainable responses in the management and rehabilitation of chronic diseases.

## Data Availability

Not applicable.
